# Th17/treg balance in Inflammatory Bowel Disease: the role of microbial, and genetic regulators in disease modulation

**DOI:** 10.3389/fcell.2026.1774790

**Published:** 2026-04-08

**Authors:** Anna Giudice, Carolina Brescia, Domenico Morano, Giuseppe Viglietto, Francesco Luzza, Rosario Amato, Rocco Spagnuolo

**Affiliations:** 1 Health Sciences Department, Magna Graecia University, Catanzaro, Italy; 2 Immuno-Genetics Lab, Department of Health Science, Medical School, University “Magna Graecia” of Catanzaro, Catanzaro, Italy; 3 Department of Experimental and Clinical Medicine, Magna Graecia University, Catanzaro, Italy

**Keywords:** gut - associated lymphoid tissues (GALT), gut microbioma, T helper 17 cells (Th17 cells), regulatory T cells (Treg cells), interleukin 17 (IL-17)

## Abstract

Inflammatory Bowel Disease (IBD) is a chronic condition characterized by persistent mucosal inflammation driven by complex interactions among the gut microbiome, host immune genetics, and cellular metabolism. Emerging evidence highlights the central role of the Th17/Treg cell balance in maintaining intestinal immune tolerance, which is tightly regulated by microbe-derived metabolites and host metabolic pathways. In IBD, microbial dysbiosis and altered metabolite profiles disrupt this equilibrium, favoring pro-inflammatory responses. Moreover, genetic variants affecting immune regulation modulate individual susceptibility and disease course. Understanding how microbiome modulation, metabolic reprogramming, and genetic predisposition converge in IBD pathogenesis opens new avenues for precision medicine. This minireview discusses recent advances in this field, emphasizing novel microbiome-targeted strategies, metabolic interventions, and personalized immunomodulatory therapies aimed at restoring Th17/Treg homeostasis. Integrating microbiome, metabolome, and immunogenetic profiling may ultimately guide tailored treatments and improve long-term outcomes in IBD.

## 1 Introduction

Inflammatory Bowel Diseases (IBD), including Crohn’s disease (CD) and Ulcerative Colitis (UC), are chronic multifactorial conditions in which dysregulation of the mucosal immune response plays a pivotal role (Hu et al., 2025; Spagnuolo et al., 2018). Under physiological conditions, the gut microbiota interacts with the host immune system through metabolic pathways, cytokine signalling, transcription factors, and receptor-mediated mechanisms. Increasing evidence indicates that an imbalance between two CD4^+^ lymphocyte-derived subsets with opposing functions, T helper 17 (Th17) and regulatory T (Treg) cells, is critical in the onset and progression of IBD. While Th17 cells promote inflammatory responses, Treg cells contribute to immune tolerance and tissue protection (Brescia et al., 2024; Zhang et al., 2024). This equilibrium is shaped by multiple regulatory pathways, including T cell receptor (TCR) and costimulatory signalling, cytokine networks, bile acids, and gut microbiome-derived metabolites (Hu et al., 2025; Yan et al., 2020; Omenetti and Pizarro, 2015; Cheng et al., 2019). The microbiome has been shown to promote T cell differentiation through the production of short-chain fatty acids and the modulation of intestinal dendritic cells. In particular, it supports a tolerogenic phenotype by promoting Treg cell development while limiting excessive Th17 cell expansion (Hu et al., 2025). Microbial metabolites can further influence this axis through epigenetic regulation, reinforcing either pro-inflammatory or regulatory programs (Luo et al., 2017). Over the years, the genetic component has proven to be important in the development of IBD. Variants affecting genes involved in Th17 and Treg regulation, such as IL-23R, CARD9, and BMI1, have been associated with altered immune responses and increased IBD risk, representing potential therapeutic targets (Audia et al., 2024; Luo et al., 2020; Gonzalez et al., 2021). Clarifying the mechanisms governing Th17/Treg dysregulation in IBD is therefore essential for advancing precision medicine strategies and developing targeted interventions aimed at restoring immune homeostasis.

## 2 Th17 and treg in intestinal inflammation: Role in IBD

Equilibrium between Th17 and Treg cells is essential for preserving mucosal integrity and preventing immune-mediated tissue damage (Hu et al., 2025; Yan et al., 2020; Zhao et al., 2021). Treg development depends on TGF-β and IL-2 whereas Th17 differentiation is driven primarily by interleukin (IL)-6 and transforming growth factor (TGF)-β. These cytokines activate intracellular signalling pathways that induce the expression of the lineage-defining transcription factors RORγt in Th17 cells and Foxp3 in Treg cells (Brescia et al., 2024). Under physiological conditions, mature Th17 cells produce limited amounts of IL-17A and IL-22, which support epithelial cell proliferation, antimicrobial peptide production, and tight junction integrity (Chen et al., 2023). These effects strengthen the mucosal barrier, prevent microbial translocation, and promote intestinal homeostasis (Liang et al., 2006; Wiche Salinas et al., 2021). IL-17A enhances intestinal epithelial cell (IEC) proliferation (Ohara et al., 2024), upregulates polymeric immunoglobulin receptor (pIgR) expression, and promotes IgA secretion, thereby improving mucosal defence (Cao et al., 2012). It also induces antimicrobial peptides involved in epithelial repair (Liang et al., 2006). In addition, IL-17A modulates immune activation by suppressing Th1-associated cytokines, such as interferon (IFN)-γ and IL-12Rβ2 (Chen et al., 2019), and promotes the polarization of M2-like macrophages, contributing to local immune regulation (Nishikawa et al., 2014). Similarly, IL-22 activates signal transducer and activator of transcription 3 (STAT3) in IECs, inducing mucin-associated gene expression and supporting goblet cell regeneration (Tawiah et al., 2018). TGF-β further reinforces immune tolerance by facilitating Th17 to Treg conversion, with Treg cells producing IL-10 and TGF-β to suppress effector T-cell responses and protect mucosal tissue (Chen et al., 2023). Genetic susceptibility and environmental factors can disrupt Th17/Treg homeostasis by altering microbiota composition and immune signalling pathways. In IBD, excessive IL-17A production amplifies mucosal inflammation, while reduced TGF-β signalling weakens regulatory control, perpetuating epithelial injury and chronic inflammation (Hu et al., 2025; Yan et al., 2020). Th17-driven inflammation promotes neutrophil recruitment and secondary tissue damage (Huang, 2021). IL-17A, alone or synergistically with tumour necrosis factor (TNF)-α, induces IECs to produce pro-inflammatory mediators, including IL-6, IL-8 (CXCL8), inducible nitric oxide synthase (iNOS), TNF-α, matrix metalloproteinases (MMPs), and granulocyte–macrophage colony-stimulating factor (GM-CSF). Co-activation of the nuclear factor kappa B (NF-κB), extracellular signal–regulated kinase (ERK) 1/2, and p38 MAPK pathways further induces IL-17C production by epithelial and goblet cells and increases CCL20 expression, thereby enhancing Th17 recruitment. Neutrophil extracellular trap (NET) formation reinforces this inflammatory circuit (Chen et al., 2023). IL-26, another Th17-associated cytokine, exacerbates inflammation by activating STAT1/3, phosphoinositide 3-kinase (PI3K)/Akt, and mitogen-activated protein kinase (MAPK) pathways, leading to increased expression of IL-6, IL-8, and MMPs in epithelial and myeloid cells (Fujii et al., 2017; Song et al., 2022). Th17 cells also exhibit marked functional plasticity, acquiring Th1- or Th2-like features depending on cytokine exposure (Brescia et al., 2024). IL-12 promotes Th17-to-Th1 conversion with enhanced IFN-γ production (Annunziato et al., 2007), whereas IL-1β, IL-4, and IL-23 favour Th2/Th17 hybrid phenotypes (Cosmi et al., 2010), contributing to chronic inflammation. Finally, Th17 cells and IL-17A are implicated in intestinal fibrosis, characterized by excessive extracellular matrix deposition and stricture formation (Biancheri et al., 2013). IL-17A levels are increased in stenotic CD tissue compared with non-stenotic regions and healthy controls (D’Alessio et al., 2022). Mechanistically, IL-17A promotes fibroblast proliferation and survival through metabolic reprogramming (Majumder et al., 2019) and induces epithelial–mesenchymal transition (EMT), increasing vimentin, Snail, and α-smooth muscle actin (α-SMA) expression while reducing E-cadherin levels (Zhang et al., 2018). In recent years, increasing evidence has indicated that dietary patterns can modulate the Th17/Treg balance by reshaping the metabolic programs that govern these 2 cell populations in IBD, thereby influencing their differentiation and functional properties (Zhang et al., 2024). Th17 cells rely predominantly on glycolysis to sustain rapid energy demands, whereas regulatory T (Treg) cells preferentially utilize oxidative phosphorylation and fatty acid β-oxidation (FAO) (Brescia et al., 2024). Excess caloric intake enhances glycolytic flux and activates the mTOR pathway, favoring Th17 differentiation and perpetuating intestinal inflammation and dysbiosis. In contrast, caloric restriction appears to exert the opposite effect by activating AMPK, which suppresses Th17 polarization while promoting Treg development. Dietary components capable of modulating this metabolic axis and consequently immune cell phenotype and function may therefore represent a promising avenue for novel therapeutic strategies in IBD (Zhang et al., 2024). Notably, both ketogenic and Mediterranean dietary patterns have been shown to reduce colonic Th17 cell frequencies while promoting a more favorable gut microbial configuration (Hu et al., 2025; Bertin et al., 2026; Ang et al., 2020). Microbiota-derived metabolites further act as critical regulators of the Th17/Treg equilibrium within the intestinal mucosa. Polyamines generated through host amino acid metabolism (e.g., arginine), synthesized by commensal bacteria from amino acids or free nitrogen, or directly obtained through the diet—play a significant role in maintaining this balance (Carriche et al., 2020; Fan et al., 2025; Niu et al., 2025). In humans, the principal polyamines include putrescine, spermidine, and cadaverine. Beyond their well-established contribution to epithelial turnover and barrier integrity, polyamines actively shape immune responses by influencing macrophage polarization and T cell activation (Fan et al., 2025). Among them, spermidine has emerged as a potent activator of the tyrosine phosphatase PTPN2, thereby attenuating IFN-γ–driven inflammation through modulation of the STAT1/STAT3 signaling pathways. Through the induction of autophagy, spermidine promotes Treg differentiation and expansion within the lamina propria, supports mucosal homeostasis, and reduces the expression of pro-inflammatory mediators. Collectively, these effects contribute to the restoration of intestinal immune equilibrium (Carriche et al., 2020; Fan et al., 2025). In parallel, metabolomic profiling has provided further insights into immune-metabolic dysregulation in IBD. A non-targeted fecal metabolomics study identified a significant reduction in N-acetylglutamine (NAG) levels in patients with CD compared with healthy controls. *In vitro* experiments demonstrated that N-acetylglutamine modulates CD4^+^ T cell differentiation by promoting Treg development while restraining pathogenic Th17 polarization. These findings suggest that N-acetylglutamine may hold therapeutic potential in re-establishing the disrupted Th17/Treg balance characteristic of IBD (Han et al., 2026).

## 3 The interplay between gut microbiota derived signals and the Th17/Treg axis in intestinal immune regulation

**FIGURE 1:**
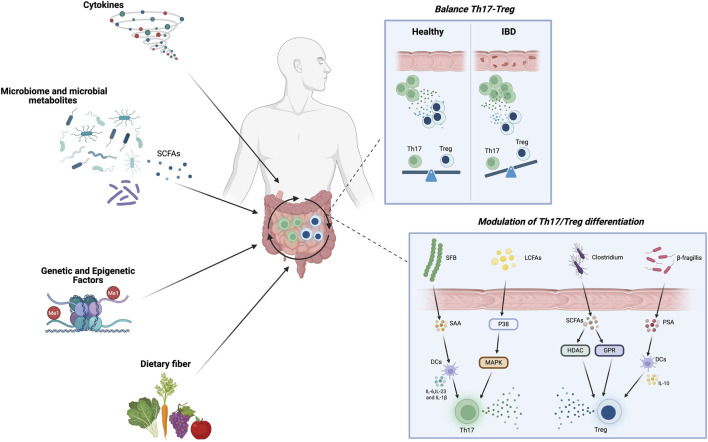
Regulation of the Th17/Treg axis in the intestinal microenvironment. Multiple factors regulate the delicate balance between Th17 and Treg cells within the intestinal mucosa, and disruption of this equilibrium represents a central immunopathogenic feature of Inflammatory Bowel Disease (IBD). Abbreviations: SFB: Segmented Filamentous Bacteria; SCFAs: Short-chain fatty acids; β-fragilis: Bacteroides fragilis; SAA: serum amyloid A; p-38: p38 mitogen-activated kinase inhibitor; PSA: Polysaccharide A; DC: dendritic cell; MAPK: mitogen-activated protein kinase; HDAC: histone deacetylase; GPR: G protein–coupled receptors; Treg cell: regulatory cell; Th17 cell: T helper type 17 cell. Created in BioRender. Brescia, C. (2026) https://BioRender.com/rlg13pg.

The gut microbiota is a key regulator of intestinal immune homeostasis and plays a central role in controlling Th17 and Treg cell differentiation and function (Omenetti and Pizarro, 2015). Commensal taxa, including Firmicutes such as *Clostridium* clusters IV and XIVa and Faecalibacterium prausnitzii, as well as Bifidobacterium spp., support tolerogenic pathways that promote Foxp3^+^ Treg differentiation. In contrast, pathobionts such as segmented filamentous bacteria (SFB) and *Fusobacterium* nucleatum activate pro-inflammatory circuits that favour Th17 cell expansion (Figure 1). The balance between these opposing microbial signals shapes the intestinal immune baseline and critically influences susceptibility to IBD (Hu et al., 2025).

### 3.1 Mechanism promoting regulatory T Cells (treg)

The induction of regulatory T (Treg) cells is primarily orchestrated by short-chain fatty acids (SCFAs) including butyrate, acetate, and propionate produced predominantly by members of the phylum Firmicutes during the fermentation of dietary fibers. These metabolites promote immune tolerance through activation of G protein–coupled receptors (GPCRs), such as GPR43 and GPR109A, and through modulation of the mTOR signaling pathway, thereby enhancing Treg differentiation (Hu et al., 2025; Zhao et al., 2018). In addition, SCFAs stimulate intestinal epithelial cells to secrete TGF-β1, which, in concert with tolerogenic dendritic cells (DCs), supports the expansion of colonic Tregs (Figure 1). The clinical relevance of this axis is underscored in patients with IBD, in whom depletion of SCFA-producing taxa particularly *Clostridium* clusters IV and XIVa and Faecalibacterium prausnitzii results in reduced SCFA bioavailability, impaired Treg generation, and the perpetuation of chronic mucosal inflammation (Li et al., 2025). Beyond the SCFA pathway, this regulatory network is further reinforced by microbial tryptophan and bile acid metabolism. Microbial catabolism of tryptophan generates indole derivatives that activate the aryl hydrocarbon receptor (AhR), thereby promoting Foxp3 expression and restraining Th17-driven inflammation, effectively linking microbial metabolic activity to the maintenance of immune tolerance. Similarly, secondary bile acids contribute to this immunoregulatory circuit by activating nuclear receptors such as the farnesoid X receptor (FXR) and pregnane X receptor (PXR), which induce Treg-associated transcriptional programs while suppressing pro-inflammatory cytokine production (Calvo-Barreiro et al., 2023). The seminal study by Atarashi et al. further demonstrated that spore-forming Clostridia are essential for the expansion of colonic Foxp3^+^ Tregs (Atarashi et al., 2011). These commensals confer protection against experimental colitis by stimulating epithelial-derived TGF-β and facilitating DC-mediated differentiation of IL-10 producing Tregs. Collectively, these interconnected pathways delineate an integrated microbiota–immune axis, in which commensal bacteria such as *Clostridium* spp. and their metabolic by-products cooperate to preserve immune tolerance at the intestinal mucosal barrier (Luo et al., 2017; Li et al., 2025).

### 3.2 Mechanism promoting TH17 differentiations

Pathogenic and opportunistic bacteria can shift immune homeostasis toward Th17-dominated responses by activating pattern-recognition receptors (PRRs) on antigen-presenting cells. Engagement of Toll-like receptor 9 (TLR9) by bacterial DNA promotes Th17 differentiation while suppressing Treg cell development. Similarly, activation of TLR2 and TLR4 induces the production of interleukin IL-6 and IL-23, thereby enhancing Th17 expansion through STAT3 signalling (Cheng et al., 2019). SFB are among the most potent inducers of Th17 cells; their close adherence to intestinal epithelial cells stimulates serum amyloid A production, which activates dendritic cells to secrete IL-6 and IL-23, driving Th17 differentiation *via* the IL-23/STAT3 and transforming growth factor (TGF)-β/Smad pathways (Figure 1). While these responses are beneficial for host defence, their persistent activation contributes to chronic inflammation in IBD (Hu et al., 2025; Chen et al., 2023). Lipid-derived metabolites further modulate this balance. Long-chain fatty acids promote Th1/Th17 polarization through activation of the p38 mitogen-activated protein kinase (MAPK) pathway, whereas SCFAs exert opposing effects by enhancing Treg proliferation. Together, these findings highlight how microbial and dietary metabolic inputs converge to shape the Th17/Treg equilibrium in the intestinal mucosa (Hu et al., 2025; Zhang et al., 2024; Yan et al., 2020).

### 3.3 Gut microbiota as a therapeutic target for IBD

**FIGURE 2:**
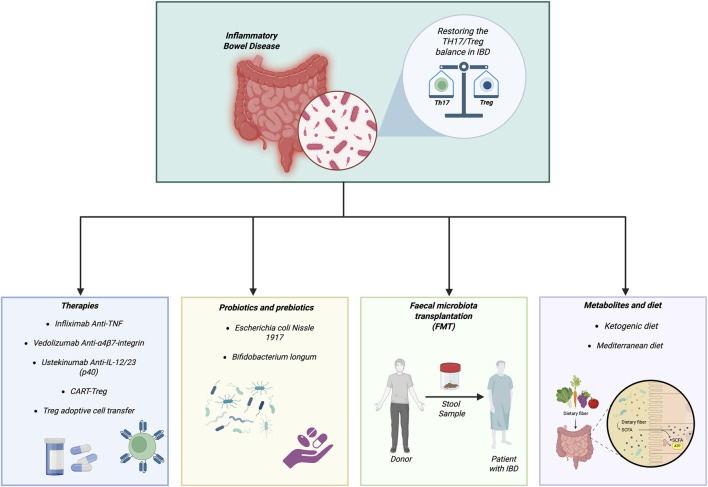
Therapeutic strategies for restoring Th17/Treg balance in IBD. The various therapeutic strategies adopted or still being tested that aim to reduce chronic intestinal inflammation in IBD by restoring the Th17/Treg balance. Created in BioRender. Brescia, C. (2026) https://BioRender.com/8oy2i51.

Clinical evidence increasingly supports the concept that targeted interventions including selected probiotics, fecal microbiota transplantation (FMT), and dietary modulation can restore immunological homeostasis in IBD by enhancing Treg-mediated responses while restraining Th17-driven inflammation (Figure 2). Several reports indicate that specific probiotic strains contribute to disease control by reshaping the intestinal immune landscape. In clinical practice, *E. coli* (notably the Nissle 1917 strain) has emerged as a promising probiotic option in ulcerative colitis (UC). A double-blind, placebo-controlled trial demonstrated that live *Escherichia coli* preparations were comparable to mesalamine in maintaining remission over a 12-month follow-up period, thus representing a potential alternative to conventional aminosalicylate therapy (Figure 2) (Scaldaferri et al., 2016). Beyond *E. coli*, lactic acid bacteria such as *Lactobacillus* rhamnosus GG have been shown to reduce pro-inflammatory cytokine production and improve colonic histological scores, effectively preventing colitis recurrence in antibiotic-treated experimental models (Li et al., 2023; Si et al., 2025). These effects are frequently mediated through reinforcement of regulatory pathways; for example, Bifidobacterium longum has been reported to expand Treg populations, enhance IL-10 production, and strengthen mucosal immune resilience (Yao et al., 2021). Nutrient intake remains a primary determinant of gut microbiota composition and mucosal immunity (Zhang, 2022). Dietary patterns profoundly influence the Th17/Treg equilibrium through interconnected metabolic and microbial mechanisms. High salt intake, for instance, promotes Th17 polarization by reducing the survival of protective *Lactobacillus* species (Wilck et al., 2017), whereas excessive dietary sugar amplifies inflammation by increasing IL-1β production and Th17 cell frequencies (Ma et al., 2022). Microbiota colonization and transplantation studies further underscore the central role of microbial diversity in maintaining immune tolerance. The transfer of defined human *Clostridium* strains or multi-strain consortia into germ-free mice markedly expands colonic RORγt^+^ Treg cells (Kedmi et al., 2022; Atarashi et al., 2013). In clinical settings, FMT has demonstrated efficacy in inducing endoscopic remission in active UC, particularly when high-dose, multi-donor protocols are employed (Figure 2) (Hsu et al., 2023). The importance of adopting an ecosystem-based therapeutic perspective is highlighted by differential outcomes observed with biologic agents. While selective IL-17A inhibition (e.g., secukinumab) may paradoxically exacerbate CD by compromising mucosal barrier integrity and amplifying Th1 responses (Dai et al., 2025), upstream blockade of the IL-12/23 p40 subunit with ustekinumab effectively sustains remission (Feagan et al., 2016). Indeed, biologic therapies for IBD including anti-TNFα agents (*infliximab* and *adalimumab*), *vedolizumab*, and *ustekinumab* are associated with significant remodeling of the gut microbiota, a process closely linked to rebalancing the Th17/Treg axis (Figure 2) (Pu et al., 2022). Anti-TNFα therapy has been shown to increase α-diversity, reduce pro-inflammatory Proteobacteria, and promote the expansion of SCFA-producing taxa such as *Faecalibacterium prausnitzii*, *Clostridiales,* Lachnospiraceae, and *Anaerostipes*. These microbial shifts become evident in clinical responders after several weeks to months of treatment and correlate strongly with endoscopic remission (Pu et al., 2022). *Vedolizumab*, by targeting the α4β7 integrin and limiting lymphocyte trafficking to the gut, similarly enhances beneficial taxa including *Roseburia inulinivorans* and *Bifidobacterium longum* (Feagan et al., 2013). *Ustekinumab* further supports microbial restoration by increasing *Faecalibacterium* and *Bacteroides populations* (Danese et al., 2015). Given the pivotal role of the IL-23/IL-23R axis in IBD pathogenesis, innovative strategies are now being explored. Recent studies are investigating the engineering of Treg cells expressing a chimeric antigen receptor (CAR) directed against IL-23R, with the aim of generating IL-23R–CAR-Treg cells for CD therapy (Figure 2) (Neurath et al., 2024; Cui et al., 2024). Preclinical data indicate that adoptive transfer of autologous Treg cells expanded *ex vivo* in the presence of TGF-β ameliorates mucosal inflammation (Fantini et al., 2006). A pilot study in CD patients reported a 40% reduction in the Clinical Disease Activity Index following infusion of autologous Tregs (Figure 2) (Hsu et al., 2023; Neurath et al., 2024). Two clinical trials evaluating autologous polyclonal Treg therapy in IBD are currently ongoing (NCT04691232; NCT03185000) (Neurath et al., 2024). In parallel, several natural phytochemicals have demonstrated the capacity to modulate the Th17/Treg balance, suggesting potential as adjunctive or lead therapeutic compounds. Curcumin, derived from turmeric, inhibits Th17 differentiation and promotes Treg expansion through modulation of STAT3 and STAT5 signaling. Epigallocatechin gallate (EGCG), a major green tea polyphenol, suppresses Th17 responses while enhancing Treg development *via* HIF-1α and STAT3 pathways. Resveratrol, abundant in red fruits, favors Treg polarization over Th17 differentiation by modulating AMPK and RORγt/STAT3 signaling. Additionally, indole-3-carbinol, found in Brassicaceae vegetables, promotes Treg development through activation of the aryl hydrocarbon receptor (AhR). Collectively, these findings highlight the translational potential of phytochemicals as natural immunomodulatory agents or scaffolds for drug development in IBD (Chang et al., 2021). Overall, these bidirectional microbiota–immune interactions underscore the therapeutic relevance of baseline microbial signatures as predictive biomarkers and support the development of integrative strategies such as combining biologics with FMT or metabolic modulation to reinforce Treg dominance, restore epithelial barrier integrity, and achieve sustained remission in patients with IBD.

## 4 Genetic regulation of the Th17/Treg axis in IBD

Several studies have demonstrated that genetic alterations in pathways regulating Th17 and Treg cell development contribute to disease susceptibility in IBD. Polymorphisms affecting IL-23 and its receptor (IL-23R) can alter the Th17/Treg balance, either promoting or protecting against disease onset (Audia et al., 2024; Xu et al., 2015; Schmitt et al., 2021). Notably, specific IL-23R variants, including R381Q (rs11209026), G149R (rs76418789), and V362I (rs41313262), are associated with protection from IBD, as reduced receptor activity leads to decreased STAT3 and STAT4 phosphorylation and attenuation of downstream inflammatory pathways. These findings highlight the IL-23/IL-23R axis as a key therapeutic target (Pastras et al., 2025). Similarly, a meta-analysis identified an association between the STAT3 rs744166 variant and increased susceptibility to UC and CD, particularly in Caucasian populations (Zhang et al., 2014). Another gene critically involved in Th17/Treg regulation and IBD pathogenesis is caspase recruitment domain-containing protein 9 (CARD9), an adaptor protein essential for antifungal and antimycobacterial immunity (Luo et al., 2020). Upon activation of C-type lectin receptors, such as Dectin-1 and Dectin-2, CARD9 forms the CARD9–BCL10–MALT1 (CBM) complex, leading to activation of the ERK and NF-κB pathways (Rosenzweig et al., 2024). This signalling cascade induces cytokine and chemokine production required for T-cell activation, microbiota control, epithelial regeneration, neutrophil recruitment, and the induction of myeloid-derived suppressor cells (nMDSCs) (Vornholz et al., 2020). CARD9 regulates the production of IL-1β, IL-6, and IL-23, thereby creating a cytokine milieu that favours Th17 differentiation. Consistently, CARD9-deficient mice exhibit impaired Th17-mediated immune responses (SOKOL et al., 2013). Genetic studies have identified both risk and protective CARD9 variants: rs10870077, rs4077515, and rs10781499 are associated with increased IBD susceptibility, whereas rs141992399 and rs200735402 confer protection, likely reflecting distinct functional effects of individual variants (Luo et al., 2020). In recent years, epigenomics has emerged as a critical layer of T-cell regulation. Epigenetic mechanisms, including DNA methylation and histone post-translational modifications, regulate gene expression without altering the DNA sequence (Agarwal et al., 2020). Naïve CD4^+^ T-cell differentiation is therefore governed not only by cytokines and transcription factors, but also by epigenetic programs that determine lineage commitment, stability, and plasticity (Brescia et al., 2024). Th17 and Treg cells are particularly dependent on epigenetic control, and accumulating evidence indicates that DNA methylation, histone modifications, and microRNAs shape Th17/Treg plasticity (Luo et al., 2017). For example, Treg stability correlates with demethylation of the Treg-specific demethylated region (TSDR) within the Foxp3 locus (Floess et al., 2007). MicroRNAs, including miR-155 and miR-21, further regulate both Th17 and Treg differentiation and function (Escobar et al., 2014; Murugaiyan et al., 2015). Importantly, microbiota-derived metabolites exert profound effects on the T-cell epigenome and, consequently, on the Th17/Treg balance (Luo et al., 2017). SCFAs, including acetate, propionate, and butyrate, promote Treg differentiation by inducing Foxp3 and IL-10 expression through histone deacetylase (HDAC) inhibition, thereby contributing to host epigenome remodelling (Furusawa et al., 2013; Arpaia et al., 2013) (Figure 1). Polysaccharide A from *Bacteroides fragilis* increases Foxp3 and IL-10 expression while suppressing IL-17 production (Round and Mazmanian, 2010), whereas Bifidobacterium and *Lactobacillus* species produce folate, supporting methylation-dependent processes that influence T-cell fate (Pompei et al., 2007) (Figure 1). Notably, these microbial metabolites can modulate T-cell epigenetics both locally in the gut and systemically (Luo et al., 2017). As discussed above, additional microbiota-derived metabolites may play a pivotal role in shaping the Th17/Treg balance within the intestinal mucosa. Among these, spermidine—a bioactive polyamine—has emerged as a key immunometabolic regulator capable of modulating T cell differentiation through epigenetic mechanisms. Specifically, spermidine influences histone acetylation and methylation patterns, thereby promoting the transcription of genes associated with regulatory programs that favor Treg differentiation, while concurrently repressing pro-inflammatory gene networks driving Th17 polarization (Carriche et al., 2020). In CD, the epigenetic regulator BMI1, a component of the Polycomb repressive complex, has been shown to maintain Treg identity and prevent their conversion into pro-inflammatory phenotypes. Loss of BMI1 function contributes to disease development, underscoring its role in immune dysregulation in IBD (Gonzalez et al., 2021). Collectively, these findings demonstrate that genetic and epigenetic mechanisms converge to shape Th17/Treg plasticity and provide a mechanistic foundation for targeted therapeutic strategies aimed at restoring immune homeostasis in IBD.

## 5 Discussion

The Th17/Treg balance represents a critical immunological checkpoint in the pathogenesis of IBD, governed by a complex interplay between microbial, metabolic, and dietary factors. The gut microbiota is a fundamental regulator of the Th17/Treg axis, and in IBD it is profoundly altered by dysbiosis, which promotes immune imbalance and chronic inflammation (Omenetti and Pizarro, 2015). Microbiota-mediated regulation of Th17/Treg homeostasis involves microbial metabolites, including short-chain fatty acids (SCFAs), tryptophan derivatives, and bile acids; pattern-recognition receptor (PRR)-mediated signalling through Toll-like receptors (TLR2, TLR4, TLR9); epigenetic mechanisms such as HDAC inhibition and Foxp3-associated histone acetylation; and cytokine-driven transcriptional pathways, including TGF-β/Smad, IL-6/STAT3, IL-23/RORγt, and STAT5 signalling. Commensal bacteria preferentially activate tolerogenic pathways, such as AhR, GPR43, and STAT5 signalling, which promote Treg differentiation, whereas pathobionts trigger pro-inflammatory cascades, notably the IL-23/STAT3 axis, driving Th17 expansion (Hu et al., 2025; Yan et al., 2020; Cheng et al., 2019). Dysbiosis in IBD disrupts these regulatory networks by depleting SCFA-producing Firmicutes, expanding Th17-inducing taxa, and altering metabolic and epigenetic pathways required for immune tolerance. These changes sustain a pro-inflammatory Th17/Treg imbalance, perpetuating epithelial damage and chronic intestinal inflammation. Clinical and preclinical evidence suggests that restoring this equilibrium is achievable through targeted microbiota modulation. Probiotic strains such as *E. coli* Nissle 1917 and Bifidobacterium longum have shown efficacy in inducing IL-10 and maintaining remission (Scaldaferri et al., 2016; Yao et al., 2021), while high-dose FMT remains a robust strategy for enhancing microbial diversity and Treg frequency (Dai et al., 2025). Furthermore, nutrient intake emerges as a pivotal regulator: while excessive salt, sugar, and calories fuel Th17-driven inflammation *via* the mTOR pathway, Mediterranean and ketogenic diets, along with caloric restriction, favor a tolerogenic environment through AMPK activation (Ma et al., 2022; Shi et al., 2025). The success of biological therapies, such as *Ustekinumab*, is partly attributed to their ability to remodel the microbiota, promoting SCFA-producing taxa like Faecalibacterium prausnitzii (Feagan et al., 2016). Host genetic factors further contribute to Th17/Treg dysregulation. Multiple genes involved in T-cell differentiation, stability, and function, including IL-23R, FOXP3, RORγt, CARD9, and BMI1, have been identified as key determinants of IBD susceptibility. Specific polymorphisms, such as IL-23R rs76418789 and CARD9 rs141992399, have been associated with altered disease risk, underscoring the importance of genetic regulation of the Th17/Treg axis. In parallel, microbial-derived metabolites, including acetate, propionate, and butyrate, modulate immune cell metabolism and epigenetic programming, influencing T-cell lineage stability and function both locally and systemically (Hu et al., 2025; Luo et al., 2017). This bidirectional interaction highlights a fundamental shift in IBD management: moving from simple cytokine blockade toward ecosystem-based strategies. Future therapeutic paradigms should aim for a synergistic approach, combining biologics with nutritional and microbial interventions to achieve a stable Treg-dominant environment and durable mucosal healing. Nutritional factors further shape this balance, as dietary components and supplements can modulate cellular metabolism and immune phenotypes (Zhang et al., 2024). Based on these insights, several therapeutic strategies aimed at restoring Th17/Treg balance have been developed. These include monoclonal antibodies targeting the IL-23 pathway (Audia et al., 2024), small-molecule inhibitors of intracellular signalling pathways, including Janus kinase (JAK) inhibitors (Feagan et al., 2016), and microbiota-based interventions, including probiotic supplementation and FMT. These approaches aim to enhance Treg induction and suppress pathogenic Th17 responses through the restoration of immunomodulatory microbial metabolites, particularly SCFAs (Hu et al., 2025).
